# Strong convergence theorems for coordinatewise negatively associated random vectors in Hilbert space

**DOI:** 10.1186/s13660-018-1678-y

**Published:** 2018-04-13

**Authors:** Xiang Huang, Yongfeng Wu

**Affiliations:** 1 College of Medicine Information Engineering, Anhui University of Chinese Medicine, Hefei, China; 2 School of Mathematics and Finance, Chuzhou University, Chuzhou, China; 3 College of Mathematics and Computer Science, Tongling University, Tongling, China

**Keywords:** 60F15, Complete convergence, Complete moment convergence, Coordinatewise negatively associated, Random vectors, Hilbert space

## Abstract

In this work, some strong convergence theorems are established for weighted sums of coordinatewise negatively associated random vectors in Hilbert spaces. The results obtained in this paper improve and extend the corresponding ones of Huan et al. (Acta Math. Hung. 144(1):132–149, 2014) as well as correct and improve the corresponding one of Ko (J. Inequal. Appl. 2017:290, 2017).

## Introduction

The concept of the complete convergence was first introduced by Hsu and Robbins [3] to prove that the arithmetic mean of independent and identically distributed (i.i.d.) random variables converges completely to the expectation of the random variables. Later on, Baum and Katz [4] generalized and extended this fundamental theorem as follows.

### Theorem A

*Let* *α* *and* *r* *be real numbers such that* $r>1$, $\alpha>1/2$ *and* $\alpha r>1$ *and let* $\{ X_{n},n\geq1\}$ *be a sequence of i*.*i*.*d*. *random variables with zero mean*. *Then the following statements are equivalent*:

- $\mathbb{E}|X_{1}|^{r}<\infty$;
- $\sum_{n=1}^{\infty}n^{\alpha r-2}\mathbb{P}( \vert \sum_{k=1}^{n}X_{k} \vert >\varepsilon n^{\alpha})<\infty$;
- $\sum_{n=1}^{\infty}n^{\alpha r-2}\mathbb{P}(\sup_{k\geq n}k^{-\alpha} \vert \sum_{i=1}^{k}X_{i} \vert >\varepsilon )<\infty$.

Since the independence assumption is not reasonable in the real practice of applications in many statistical problems. This result has been extended to many classes of dependent random variables. A classical extension of independence is negative association, which was introduced by Joag-Dev and Proschan [5] as follows.

### Definition 1.1

A finite family of random variables $\{X_{i},1\leq i\leq n\}$ is said to be negatively associated (NA) if for every pair of disjoint subsets *A* and *B* of $\{1,2,\ldots,n\}$ and any real coordinatewise nondecreasing (or nonincreasing) functions $f_{1}$ on $\mathbb{R}^{A}$ and $f_{2}$ on $\mathbb{R}^{B}$,

$$\operatorname{Cov}\bigl(f_{1}(X_{i},i\in A),f_{2}(X_{j},j\in B)\bigr)\leq0, $$

whenever the covariance above exists. An infinite family of random variables is NA if every finite subfamily is NA.

There are many results based on NA random variables, we refer to Shao [6], Kuczmaszewska [7], Baek et al. [8], Kuczmaszewska and Lagodowski [9].

Let *H* be a real separable Hilbert space with the norm $\|\cdot\|$ generated by an inner product $\langle\cdot,\cdot\rangle$. Denote $X^{(j)}=\langle X,e^{(j)}\rangle$, where $\{e^{(j)},j\geq1\}$ is an orthonormal basis in *H*, and *X* is an *H*-valued random vector. Ko et al. [10] introduced the following concept of *H*-valued NA sequence.

### Definition 1.2

A sequence $\{X_{n},n\geq1\} $ of *H*-valued random vectors is said to be NA if there exists an orthonormal basis $\{e^{(j)},j\geq 1\}$ in *H* such that, for any $d\geq1$, the sequence $\{ (X_{n}^{(1)},X_{n}^{(2)},\ldots,X_{n}^{(d)}),n\geq1\}$ of $\mathbb {R}^{d}$-valued random vectors is NA.

Ko et al. [10] and Thanh [11], respectively, obtained the almost sure convergence for NA random vectors in Hilbert space. Miao [12] established the Hajeck–Renyi inequality for *H*-valued NA random vectors.

Huan et al. [1] introduced the concept of coordinatewise negative association for random vectors in Hilbert space as follows, which is more general than that of Definition 1.2.

### Definition 1.3

If for each $j\geq1$, the sequence $\{X_{n}^{(j)},n\geq1\}$ of random variables is NA, where $X_{n}^{(j)}=\langle X_{n},e^{(j)}\rangle$, then the sequence $\{X_{n},n\geq1\}$ of *H*-valued random vectors is said to be coordinatewise negatively associated (CNA).

Obviously, if a sequence of random vectors in Hilbert space is NA, it is CNA. However, generally speaking, the reverse is not true. One can see in Example 1.4 of Huan et al. [1].

Huan et al. [1] extended Theorem A from independence to the case of CNA random vectors in Hilbert space. Huan [13] extended this complete convergence result for *H*-valued CNA random vectors to the case of $1< r<2$ and $\alpha r=1$. However, the interesting case $r=1$, $\alpha r=1$ was not considered in these papers. Recently, Ko [2] extended the results of Huan et al. [1] from the complete convergence to the complete moment convergence as follows. For more details as regards the complete moment convergence, one can refer to Ko [2] and the references therein.

### Theorem B

*Let* $1\leq r<2$ *and* $\alpha r>1$. *Let* $\{X_{n},n\geq1\}$ *be a sequence of zero mean* *H*-*valued CNA random vectors*. *If* $\{X_{n},n\geq1\}$ *is coordinatewise weakly upper bounded by a random vector* *X* *satisfying* $\sum_{j=1}^{\infty}\mathbb {E}|X^{(j)}|^{r}<\infty$, *then*

$$ \sum_{n=1}^{\infty}n^{\alpha r-\alpha-2}\mathbb {E} \Biggl(\max_{1\leq k\leq n} \Biggl\Vert \sum _{i=1}^{k}X_{i} \Biggr\Vert -\varepsilon n^{\alpha} \Biggr)_{+}< \infty. $$

However, there are some mistakes in the proof of the result in the case $r=1$. In specific, the formulas $\int _{1}^{u}y^{r-2}\,dy\leq Cu^{r-1}$ of Eq. (2.7) and $\sum_{n=1}^{m}n^{\alpha r-1-\alpha}\leq Cm^{\alpha r-\alpha}$ of Eq. (2.9) in Ko [2] are wrong when $r=1$, the same problem also occurs in the proof of $I_{222}$ (see the proof of Lemma 2.5 in Ko [2]). Moreover, the interesting case $\alpha r=1$ was not considered in this paper.

In this paper, the results of the complete convergence and the complete moment convergence are established for CNA random vectors in Hilbert spaces. The results are focused on the weighted sums, which is more general than partial sums. The interesting case $\alpha r=1$ is also considered in this article. Moreover, the results of the complete moment convergence are considered with the exponent $0< q<2$ while in Theorem B only the case $q=1$ was obtained.

Recall that if $n^{-1}\sum_{i=1}^{n}\mathbb{P}(|X_{i}^{(j)}|>x)\leq C\mathbb{P}(|X^{(j)}|>x)$ for all $j\geq1$, $n\geq1$ and $x\geq0$, then the sequence $\{X_{n},n\geq1\}$ is said to be coordinatewise weakly upper bounded by *X*, where $X_{n}^{(j)}=\langle X,e^{(j)}\rangle$ and $X^{(j)}=\langle X,e^{(j)}\rangle$. Throughout the paper, let *C* be a positive constant whose value may vary in different places. Let $\log{x}=\ln\max (x,e)$ and $I(\cdot)$ be the indicator function.

## Preliminaries

In this section, we state some lemmas which will be used in the proofs of our main results.

### Lemma 2.1

(Huan et al. [1])

*Let* $\{ X_{n},n\geq1\}$ *be a sequence of* *H*-*valued CNA random vectors with zero means and* $\mathbb{E}\|X_{n}\|^{2}<\infty$ *for all* $n\geq1$. *Then*

$$ \mathbb{E}\max_{1\leq k\leq n} \Biggl\Vert \sum _{i=1}^{k}X_{i} \Biggr\Vert ^{2}\leq2\sum_{i=1}^{n}\mathbb{E} \|X_{i}\|^{2}. $$

### Lemma 2.2

(Kuczmaszewska [7])

*Let* $\{ Z_{n},n\geq1\}$ *be a sequence of random variables weakly dominated by a random variable* *Z*, *that is*, $n^{-1}\sum_{i=1}^{n}\mathbb {P}(|Z_{i}|>x)\leq C\mathbb{P}(|Z|>x)$ *for any* $x\geq0$. *Then*, *for any* $a>0$ *and* $b>0$, *there exist some positive constants* $C_{1}$ *and* $C_{2}$ *such that*

$$\begin{aligned}& n^{-1}\sum_{i=1}^{n}\mathbb {E} \vert Z_{i} \vert ^{a}I\bigl( \vert Z_{i} \vert >b\bigr)\leq C_{1}\mathbb{E} \vert Z \vert ^{a}I \bigl( \vert Z \vert >b\bigr); \\& n^{-1}\sum_{i=1}^{n}\mathbb{E} \vert X_{i} \vert ^{a}I\bigl( \vert Z_{i} \vert \leq b\bigr)\leq C_{2}\bigl[\mathbb{E} \vert Z \vert ^{a}I\bigl( \vert Z \vert \leq b\bigr)+b^{a}\mathbb{P} \bigl( \vert Z \vert >b\bigr)\bigr]. \end{aligned}$$

### Lemma 2.3

*Let* $1\leq r<2$ *and* $\alpha r\geq 1$. *Let* $\{a_{ni},1\leq i\leq n,n\geq1\}$ *be an array of real numbers such that* $\sum_{i=1}^{n}a_{ni}^{2}=O(n)$. *Let* $\{X_{n},n\geq1\}$ *be a sequence of zero mean* *H*-*valued CNA random vectors*. *Suppose that* $\{ X_{n},n\geq1\}$ *is coordinatewise weakly upper bounded by a random vector* *X*. *Assume that one of the following assumptions holds*:

(i) $\sum_{j=1}^{\infty}\mathbb{E}|X^{(j)}|^{r}<\infty$ *if* $0< q< r$;
(ii) $\sum_{j=1}^{\infty}\mathbb{E}|X^{(j)}|^{r}\log |X^{(j)}|<\infty$ *if* $q=r$;
(iii) $\sum_{j=1}^{\infty}\mathbb{E}|X^{(j)}|^{q}<\infty$ *if* $r< q<2$.

*Then*

$$ \sum_{n=1}^{\infty}n^{\alpha r-\alpha q-2} \int _{n^{\alpha q}}^{\infty}\mathbb{P} \Biggl(\max _{1\leq k\leq n} \Biggl\Vert \sum_{i=1}^{k}a_{ni}X_{i} \Biggr\Vert >t^{1/q} \Biggr)\,dt< \infty. $$

### Proof

Without loss of generality, we may assume that $a_{ni}\geq0$ for each $1\leq i\leq n$, $n\geq 1$. For any $t>0$ and each $j\geq1$, denote

$$\begin{aligned}& Y_{i}^{(j)}=-t^{1/q}I\bigl(X_{i}^{(j)}< -t^{1/q} \bigr)+X_{i}^{(j)}I\bigl( \bigl\vert X_{i}^{(j)} \bigr\vert \leq t^{1/q}\bigr)+t^{1/q}I\bigl(X_{i}^{(j)}>t^{1/q} \bigr); \\& Z_{i}^{(j)}=X_{i}^{(j)}-Y_{i}^{(j)}= \bigl(X_{i}^{(j)}+t^{1/q}\bigr)I\bigl(X_{i}^{(j)}< -t^{1/q} \bigr)+\bigl(X_{i}^{(j)}-t^{1/q}\bigr)I \bigl(X_{i}^{(j)}>t^{1/q}\bigr); \\& Y_{i}=\sum_{j=1}^{\infty}Y_{i}^{(j)}e_{j} \quad \text{and}\quad Z_{i}=\sum_{j=1}^{\infty}Z_{i}^{(j)}e_{j}. \end{aligned}$$

It is easy to obtain

$$\begin{aligned}& \sum_{n=1}^{\infty}n^{\alpha r-\alpha q-2} \int _{n^{\alpha q}}^{\infty}\mathbb{P} \Biggl(\max _{1\leq k\leq n} \Biggl\Vert \sum_{i=1}^{k}a_{ni}X_{i} \Biggr\Vert >t^{1/q} \Biggr)\,dt \\& \quad = \sum_{n=1}^{\infty}n^{\alpha r-\alpha q-2} \int_{n^{\alpha q}}^{\infty}\mathbb{P} \Biggl(\max _{1\leq i\leq n} \Biggl\Vert \sum_{i=1}^{k}a_{ni} \sum_{j=1}^{\infty}X_{i}^{(j)}e_{j} \Biggr\Vert >t^{1/q} \Biggr)\,dt \\& \quad \leq \sum_{n=1}^{\infty}n^{\alpha r-\alpha q-2} \int_{n^{\alpha q}}^{\infty}\mathbb{P} \Bigl(\max _{1\leq k\leq n}\max_{j\geq 1}\bigl|X_{i}^{(j)}\bigr|>t^{1/q} \Bigr)\,dt \\& \qquad {} +\sum_{n=1}^{\infty}n^{\alpha r-\alpha q-2} \int_{n^{\alpha q}}^{\infty}\mathbb{P} \Biggl(\max _{1\leq k\leq n} \Biggl\Vert \sum_{i=1}^{k}a_{ni} \sum_{j=1}^{\infty}Y_{i}^{(j)}e_{j} \Biggr\Vert >t^{1/q} \Biggr)\,dt \\& \quad \leq \sum_{n=1}^{\infty}n^{\alpha r-\alpha q-2} \int_{n^{\alpha q}}^{\infty}\sum_{j=1}^{\infty} \sum_{i=1}^{n}\mathbb{P} \bigl( \bigl\vert X_{i}^{(j)} \bigr\vert >t^{1/q} \bigr)\,dt \\& \qquad {} +\sum_{n=1}^{\infty}n^{\alpha r-\alpha q-2} \int_{n^{\alpha q}}^{\infty}\mathbb{P} \Biggl(\max _{1\leq k\leq n} \Biggl\Vert \sum_{i=1}^{k}a_{ni}Y_{i} \Biggr\Vert >t^{1/q} \Biggr)\,dt \\& \quad := J_{1}+J_{2}. \end{aligned}$$

By Lemma 2.2, we derive that

$$\begin{aligned} J_{1} \leq&C\sum_{j=1}^{\infty}\sum _{n=1}^{\infty }n^{\alpha r-\alpha q-1} \int_{n^{\alpha q}}^{\infty}\mathbb{P} \bigl( \bigl\vert X^{(j)} \bigr\vert >t^{1/q} \bigr)\,dt \\ \leq&C\sum_{j=1}^{\infty}\sum _{n=1}^{\infty}n^{\alpha r-\alpha q-1}\mathbb{E} \bigl\vert X^{(j)} \bigr\vert ^{q}I\bigl( \bigl\vert X^{(j)} \bigr\vert >n^{\alpha}\bigr) \\ =&C\sum_{j=1}^{\infty}\sum _{m=1}^{\infty}\mathbb {E} \bigl\vert X^{(j)} \bigr\vert ^{q}I\bigl(m^{\alpha}< \bigl\vert X^{(j)} \bigr\vert \leq(m+1)^{\alpha}\bigr)\sum_{n=1}^{m}n^{\alpha r-\alpha q-1}. \end{aligned}$$

Therefore, if $q< r$,

$$ J_{1}\leq C\sum_{j=1}^{\infty}\sum _{m=1}^{\infty }m^{\alpha r-\alpha q}\mathbb{E} \bigl\vert X^{(j)} \bigr\vert ^{q}I\bigl(m^{\alpha }< \bigl\vert X^{(j)} \bigr\vert \leq(m+1)^{\alpha}\bigr)\leq C\sum _{j=1}^{\infty}\mathbb {E} \bigl\vert X^{(j)} \bigr\vert ^{r}< \infty; $$

if $q=r$,

$$ J_{1}\leq C\sum_{j=1}^{\infty}\sum _{m=1}^{\infty }\log{m}\mathbb{E} \bigl\vert X^{(j)} \bigr\vert ^{r}I\bigl(m^{\alpha}< \bigl\vert X^{(j)} \bigr\vert \leq (m+1)^{\alpha}\bigr)\leq C\sum _{j=1}^{\infty}\mathbb{E} \bigl\vert X^{(j)} \bigr\vert ^{r}\log \bigl\vert X^{(j)} \bigr\vert < \infty; $$

and if $r< q<2$,

$$ J_{1}\leq C\sum_{j=1}^{\infty}\sum _{m=1}^{\infty }\mathbb{E} \bigl\vert X^{(j)} \bigr\vert ^{q}I\bigl(m^{\alpha}< \bigl\vert X^{(j)} \bigr\vert \leq(m+1)^{\alpha }\bigr)\leq C\sum _{j=1}^{\infty}\mathbb{E} \bigl\vert X^{(j)} \bigr\vert ^{q}< \infty. $$

To estimate $J_{2}$, we first show that

$$ \sup_{t\geq n^{\alpha q}}t^{-1/q}\max_{1\leq k\leq n} \Biggl\Vert \sum_{i=1}^{k}a_{ni} \mathbb{E}Y_{i} \Biggr\Vert \rightarrow 0\quad \text{as }n\rightarrow \infty. $$

Actually, noting by the Hölder inequality that $\sum_{i=1}^{n}a_{ni}=O(n)$, we have by the zero mean assumption

$$\begin{aligned} \sup_{t\geq n^{\alpha q}}t^{-1/q}\max_{1\leq k\leq n} \Biggl\Vert \sum_{i=1}^{k}a_{ni} \mathbb{E}Y_{i} \Biggr\Vert =&\sup_{t\geq n^{\alpha q}}t^{-1/q} \max_{1\leq k\leq n} \Biggl\Vert \sum_{i=1}^{k}a_{ni} \mathbb{E}Z_{i} \Biggr\Vert \\ \leq&n^{-\alpha}\sup_{t\geq n^{\alpha q}}\sum _{j=1}^{\infty}\sum_{i=1}^{n}a_{ni} \mathbb{E} \bigl\vert X_{i}^{(j)} \bigr\vert I\bigl( \bigl\vert X_{i}^{(j)} \bigr\vert >t^{1/q}\bigr) \\ \leq&n^{-\alpha}\sum_{j=1}^{\infty}\sum _{i=1}^{n}a_{ni}\mathbb {E} \bigl\vert X_{i}^{(j)} \bigr\vert I\bigl( \bigl\vert X_{i}^{(j)} \bigr\vert >n^{\alpha}\bigr) \\ \leq&Cn^{1-\alpha}\sum_{j=1}^{\infty} \mathbb {E} \bigl\vert X^{(j)} \bigr\vert I\bigl( \bigl\vert X^{(j)} \bigr\vert >n^{\alpha}\bigr) \\ \leq&Cn^{1-\alpha r}\sum_{j=1}^{\infty} \mathbb {E} \bigl\vert X^{(j)} \bigr\vert ^{r}I\bigl( \bigl\vert X^{(j)} \bigr\vert >n^{\alpha}\bigr)\rightarrow0\quad \text{as }n\rightarrow\infty, \end{aligned}$$

provided that $\alpha r>1$. If $\alpha r=1$, the conclusion above remains true by the dominated convergence theorem. Therefore, when *n* is large enough, for any $t\geq n^{\alpha q}$,

1 $$ \max_{1\leq k\leq n} \Biggl\Vert \sum_{i=1}^{k}a_{ni} \mathbb{E}Y_{i} \Biggr\Vert \leq t^{1/q}/2. $$

Since $\{a_{ni}Y_{i}^{(j)},1\leq i\leq n,n\geq1\}$ is NA for any $j\geq1$, $\{a_{ni}(Y_{i}-\mathbb{E}Y_{i}),1\leq i\leq n,n\geq1\}$ is CNA. Hence, by the Markov inequality, Lemmas 2.1 and 2.2, $\sum_{i=1}^{n}a_{ni}^{2}=O(n)$ and (1),

$$\begin{aligned} J_{2} \leq&C\sum_{n=1}^{\infty}n^{\alpha r-\alpha q-2} \int_{n^{\alpha q}}^{\infty}\mathbb{P} \Biggl(\max _{1\leq k\leq n} \Biggl\Vert \sum_{i=1}^{k}a_{ni}(Y_{i}- \mathbb{E}Y_{i}) \Biggr\Vert >t^{1/q}/2 \Biggr)\,dt \\ \leq&C\sum_{n=1}^{\infty}n^{\alpha r-\alpha q-2} \int_{n^{\alpha q}}^{\infty}t^{-2/q}\mathbb{E} \Biggl(\max _{1\leq k\leq n} \Biggl\Vert \sum_{i=1}^{k}a_{ni}(Y_{i}- \mathbb{E}Y_{i}) \Biggr\Vert \Biggr)^{2}\,dt \\ \leq&C\sum_{n=1}^{\infty}n^{\alpha r-\alpha q-2} \int_{n^{\alpha q}}^{\infty}t^{-2/q}\sum _{i=1}^{n}a_{ni}^{2}\mathbb{E}\| Y_{i}-\mathbb{E}Y_{i}\|^{2}\,dt \\ \leq&C\sum_{n=1}^{\infty}n^{\alpha r-\alpha q-2} \int_{n^{\alpha q}}^{\infty}t^{-2/q}\sum _{i=1}^{n}a_{ni}^{2}\mathbb{E} \|Y_{i}\| ^{2}\,dt \\ \leq&C\sum_{j=1}^{\infty}\sum _{n=1}^{\infty}n^{\alpha r-\alpha q-2} \int_{n^{\alpha q}}^{\infty}t^{-2/q}\sum _{i=1}^{n}a_{ni}^{2}\mathbb{E} \bigl\vert Y_{i}^{(j)} \bigr\vert ^{2}\,dt \\ \leq&C\sum_{j=1}^{\infty}\sum _{n=1}^{\infty}n^{\alpha r-\alpha q-2} \int_{n^{\alpha q}}^{\infty}\sum_{i=1}^{n}a_{ni}^{2} \mathbb {P}\bigl( \bigl\vert X^{(j)} \bigr\vert >t^{1/q} \bigr)\,dt \\ &{}+C\sum_{j=1}^{\infty}\sum _{n=1}^{\infty}n^{\alpha r-\alpha q-2} \int_{n^{\alpha q}}^{\infty}t^{-2/q}\sum _{i=1}^{n}a_{ni}^{2}\mathbb{E} \bigl\vert X^{(j)} \bigr\vert ^{2}I\bigl( \bigl\vert X^{(j)} \bigr\vert \leq t^{1/q}\bigr)\,dt \\ \leq&C\sum_{j=1}^{\infty}\sum _{n=1}^{\infty}n^{\alpha r-\alpha q-1} \int_{n^{\alpha q}}^{\infty}\mathbb{P}\bigl( \bigl\vert X^{(j)} \bigr\vert >t^{1/q}\bigr)\,dt \\ &{}+C\sum_{j=1}^{\infty}\sum _{n=1}^{\infty}n^{\alpha r-\alpha q-1} \int_{n^{\alpha q}}^{\infty}t^{-2/q}\mathbb {E} \bigl\vert X^{(j)} \bigr\vert ^{2}I\bigl( \bigl\vert X^{(j)} \bigr\vert \leq t^{1/q}\bigr)\,dt \\ =:&J_{21}+J_{22}. \end{aligned}$$

Similar to the proof of $J_{1}<\infty$, we have $J_{21}<\infty$. Finally, we will estimate $J_{22}$. By some standard calculation, we have

$$\begin{aligned} J_{22} \leq&C\sum_{j=1}^{\infty}\sum _{n=1}^{\infty }n^{\alpha r-\alpha q-1}\sum _{m=n}^{\infty} \int_{m^{\alpha q}}^{(m+1)^{\alpha q}}t^{-2/q}\mathbb{E} \bigl\vert X^{(j)} \bigr\vert ^{2}I\bigl( \bigl\vert X^{(j)} \bigr\vert \leq t^{1/q}\bigr)\,dt \\ \leq&C\sum_{j=1}^{\infty}\sum _{n=1}^{\infty}n^{\alpha r-\alpha q-1}\sum _{m=n}^{\infty}m^{\alpha q-2\alpha-1}\mathbb {E} \bigl\vert X^{(j)} \bigr\vert ^{2}I\bigl( \bigl\vert X^{(j)} \bigr\vert \leq(m+1)^{\alpha}\bigr) \\ =&C\sum_{j=1}^{\infty}\sum _{m=1}^{\infty}m^{\alpha q-2\alpha -1}\mathbb{E} \bigl\vert X^{(j)} \bigr\vert ^{2}I\bigl( \bigl\vert X^{(j)} \bigr\vert \leq(m+1)^{\alpha}\bigr)\sum _{n=1}^{m}n^{\alpha r-\alpha q-1}. \end{aligned}$$

Since the upper bound of $\sum_{n=1}^{m}n^{\alpha r-\alpha q-1}$ is different by choosing different values of *q*, we consider the following three cases. If $q< r$, we have

$$\begin{aligned} J_{22} \leq&C\sum_{j=1}^{\infty}\sum _{m=1}^{\infty }m^{\alpha r-2\alpha-1}\mathbb{E} \bigl\vert X^{(j)} \bigr\vert ^{2}I\bigl( \bigl\vert X^{(j)} \bigr\vert \leq (m+1)^{\alpha}\bigr) \\ \leq&C\sum_{j=1}^{\infty}\sum _{m=1}^{\infty}m^{\alpha r-2\alpha -1}\mathbb{E} \bigl\vert X^{(j)} \bigr\vert ^{2}I\bigl( \bigl\vert X^{(j)} \bigr\vert \leq1\bigr) \\ &{}+C\sum_{j=1}^{\infty}\sum _{m=1}^{\infty}m^{\alpha r-2\alpha -1}\sum _{l=1}^{m}\mathbb{E} \bigl\vert X^{(j)} \bigr\vert ^{2}I\bigl(l^{\alpha}< \bigl\vert X^{(j)} \bigr\vert \leq (l+1)^{\alpha}\bigr) \\ \leq&C\sum_{j=1}^{\infty}\mathbb{E} \bigl\vert X^{(j)} \bigr\vert ^{r}+C\sum _{j=1}^{\infty}\sum_{l=1}^{\infty} \mathbb {E} \bigl\vert X^{(j)} \bigr\vert ^{2}I \bigl(l^{\alpha}< \bigl\vert X^{(j)} \bigr\vert \leq(l+1)^{\alpha}\bigr)\sum_{m=l}^{\infty}m^{\alpha r-2\alpha-1} \\ \leq&C\sum_{j=1}^{\infty}\mathbb{E} \bigl\vert X^{(j)} \bigr\vert ^{r}+C\sum _{j=1}^{\infty}\sum_{l=1}^{\infty}l^{\alpha r-2\alpha} \mathbb {E} \bigl\vert X^{(j)} \bigr\vert ^{2}I \bigl(l^{\alpha}< \bigl\vert X^{(j)} \bigr\vert \leq(l+1)^{\alpha}\bigr) \\ \leq&C\sum_{j=1}^{\infty}\mathbb{E} \bigl\vert X^{(j)} \bigr\vert ^{r}< \infty; \end{aligned}$$

if $q=r$, we have

$$\begin{aligned} J_{22} \leq&C\sum_{j=1}^{\infty}\sum _{m=1}^{\infty }m^{\alpha r-2\alpha-1}\log m \mathbb{E} \bigl\vert X^{(j)} \bigr\vert ^{2}I\bigl( \bigl\vert X^{(j)} \bigr\vert \leq (m+1)^{\alpha}\bigr) \\ \leq&C\sum_{j=1}^{\infty}\sum _{m=1}^{\infty}m^{\alpha r-2\alpha -1}\log m \mathbb{E} \bigl\vert X^{(j)} \bigr\vert ^{2}I\bigl( \bigl\vert X^{(j)} \bigr\vert \leq1\bigr) \\ &{}+C\sum_{j=1}^{\infty}\sum _{m=1}^{\infty}m^{\alpha r-2\alpha -1}\log m \sum _{l=1}^{m}\mathbb{E} \bigl\vert X^{(j)} \bigr\vert ^{2}I\bigl(l^{\alpha }< \bigl\vert X^{(j)} \bigr\vert \leq(l+1)^{\alpha}\bigr) \\ \leq&C\sum_{j=1}^{\infty}\mathbb{E} \bigl\vert X^{(j)} \bigr\vert ^{r}+C\sum _{j=1}^{\infty}\sum_{l=1}^{\infty} \mathbb {E} \bigl\vert X^{(j)} \bigr\vert ^{2}I \bigl(l^{\alpha}< \bigl\vert X^{(j)} \bigr\vert \leq(l+1)^{\alpha}\bigr)\sum_{m=l}^{\infty}m^{\alpha r-2\alpha-1} \log m \\ \leq&C\sum_{j=1}^{\infty}\mathbb{E} \bigl\vert X^{(j)} \bigr\vert ^{r}+C\sum _{j=1}^{\infty}\sum_{l=1}^{\infty}l^{\alpha r-2\alpha} \log l \mathbb{E} \bigl\vert X^{(j)} \bigr\vert ^{2}I \bigl(l^{\alpha}< \bigl\vert X^{(j)} \bigr\vert \leq(l+1)^{\alpha}\bigr) \\ \leq&C\sum_{j=1}^{\infty}\mathbb{E} \bigl\vert X^{(j)} \bigr\vert ^{r}+C\sum _{j=1}^{\infty}\mathbb{E} \bigl\vert X^{(j)} \bigr\vert ^{r}\log \bigl\vert X^{(j)} \bigr\vert < \infty; \end{aligned}$$

and if $r< q<2$, we have

$$\begin{aligned} J_{22} \leq&C\sum_{j=1}^{\infty}\sum _{m=1}^{\infty }m^{\alpha q-2\alpha-1}\mathbb{E} \bigl\vert X^{(j)} \bigr\vert ^{2}I\bigl( \bigl\vert X^{(j)} \bigr\vert \leq (m+1)^{\alpha}\bigr) \\ \leq&C\sum_{j=1}^{\infty}\sum _{m=1}^{\infty}m^{\alpha q-2\alpha -1}\mathbb{E} \bigl\vert X^{(j)} \bigr\vert ^{2}I\bigl( \bigl\vert X^{(j)} \bigr\vert \leq1\bigr) \\ &{}+C\sum_{j=1}^{\infty}\sum _{m=1}^{\infty}m^{\alpha q-2\alpha -1}\sum _{l=1}^{m}\mathbb{E} \bigl\vert X^{(j)} \bigr\vert ^{2}I\bigl(l^{\alpha}< \bigl\vert X^{(j)} \bigr\vert \leq (l+1)^{\alpha}\bigr) \\ \leq&C\sum_{j=1}^{\infty}\mathbb{E} \bigl\vert X^{(j)} \bigr\vert ^{q}+C\sum _{j=1}^{\infty}\sum_{l=1}^{\infty} \mathbb {E} \bigl\vert X^{(j)} \bigr\vert ^{2}I \bigl(l^{\alpha}< \bigl\vert X^{(j)} \bigr\vert \leq(l+1)^{\alpha}\bigr)\sum_{m=l}^{\infty}m^{\alpha q-2\alpha-1} \\ \leq&C\sum_{j=1}^{\infty}\mathbb{E} \bigl\vert X^{(j)} \bigr\vert ^{q}+C\sum _{j=1}^{\infty}\sum_{l=1}^{\infty}l^{\alpha q-2\alpha} \mathbb {E} \bigl\vert X^{(j)} \bigr\vert ^{2}I \bigl(l^{\alpha}< \bigl\vert X^{(j)} \bigr\vert \leq(l+1)^{\alpha}\bigr) \\ \leq&C\sum_{j=1}^{\infty}\mathbb{E} \bigl\vert X^{(j)} \bigr\vert ^{q}< \infty. \end{aligned}$$

Consequently, the proof of the lemma is completed. □

## Main results and discussion

In this section, we will present the main results and their proofs as follows.

### Theorem 3.1

*Let* $1\leq r<2$ *and* $\alpha r\geq 1$. *Let* $\{a_{ni},1\leq i\leq n,n\geq1\}$ *be an array of real numbers such that* $\sum_{i=1}^{n}a_{ni}^{2}=O(n)$. *Let* $\{X_{n},n\geq1\}$ *be sequence of zero mean* *H*-*valued CNA random vectors*. *If* $\{X_{n},n\geq 1\}$ *is coordinatewise weakly upper bounded by a random vector* *X*, *then* $\sum_{j=1}^{\infty}\mathbb{E}|X^{(j)}|^{r}<\infty$ *implies for any* $\varepsilon>0$ *that*

$$ \sum_{n=1}^{\infty}n^{\alpha r-2}\mathbb{P} \Biggl(\max_{1\leq k\leq n} \Biggl\Vert \sum _{i=1}^{k}a_{ni}X_{i} \Biggr\Vert >\varepsilon n^{\alpha} \Biggr)< \infty. $$

### Proof

Without loss of generality, we may assume that $a_{ni}\geq0$ for each $1\leq i\leq n$, $n\geq1$. For each $n\geq 1$ and each $j\geq1$, denote

$$\begin{aligned}& U_{i}^{(j)}=-n^{\alpha}I\bigl(X_{i}^{(j)}< -n^{\alpha } \bigr)+X_{i}^{(j)}I\bigl( \bigl\vert X_{i}^{(j)} \bigr\vert \leq n^{\alpha}\bigr)+n^{\alpha }I\bigl(X_{i}^{(j)}>n^{\alpha} \bigr); \\& V_{i}^{(j)}=X_{i}^{(j)}-V_{i}^{(j)}= \bigl(X_{i}^{(j)}+n^{\alpha }\bigr)I\bigl(X_{i}^{(j)}< -n^{\alpha} \bigr)+\bigl(X_{i}^{(j)}-n^{\alpha }\bigr)I \bigl(X_{i}^{(j)}>n^{\alpha}\bigr); \\& U_{i}=\sum_{j=1}^{\infty}U_{i}^{(j)}e_{j} \quad \text{and}\quad V_{i}=\sum_{j=1}^{\infty}V_{i}^{(j)}e_{j}. \end{aligned}$$

It is easy to obtain

$$\begin{aligned}& \sum_{n=1}^{\infty}n^{\alpha r-2}\mathbb{P} \Biggl(\max_{1\leq k\leq n} \Biggl\Vert \sum _{i=1}^{k}a_{ni}X_{i} \Biggr\Vert >\varepsilon n^{\alpha} \Biggr) \\& \quad = \sum_{n=1}^{\infty}n^{\alpha r-2} \mathbb{P} \Biggl(\max_{1\leq k\leq n} \Biggl\Vert \sum _{i=1}^{k}a_{ni}\sum _{j=1}^{\infty }X_{i}^{(j)}e_{j} \Biggr\Vert >\varepsilon n^{\alpha} \Biggr) \\& \quad \leq \sum_{n=1}^{\infty}n^{\alpha r-2} \mathbb{P} \Bigl(\max_{1\leq i\leq n}\max_{j\geq1}\bigl|X_{i}^{(j)}\bigr|> n^{\alpha} \Bigr) \\& \qquad {} +\sum_{n=1}^{\infty}n^{\alpha r-2} \mathbb{P} \Biggl(\max_{1\leq k\leq n} \Biggl\Vert \sum _{i=1}^{k}a_{ni}\sum _{j=1}^{\infty }U_{i}^{(j)}e_{j} \Biggr\Vert >\varepsilon n^{\alpha} \Biggr) \\& \quad \leq \sum_{n=1}^{\infty}n^{\alpha r-2} \sum_{j=1}^{\infty}\sum _{i=1}^{n}\mathbb{P} \bigl( \bigl\vert X_{i}^{(j)} \bigr\vert > n^{\alpha} \bigr) \\& \qquad {} +\sum_{n=1}^{\infty}n^{\alpha r-2} \mathbb{P} \Biggl(\max_{1\leq k\leq n} \Biggl\Vert \sum _{i=1}^{k}a_{ni}U_{i} \Biggr\Vert >\varepsilon n^{\alpha} \Biggr) \\& \quad := I_{1}+I_{2}. \end{aligned}$$

By weakly upper bounded assumption and Lemma 2.2, we have

$$\begin{aligned} I_{1} \leq&C\sum_{j=1}^{\infty}\sum _{n=1}^{\infty }n^{\alpha r-1}\mathbb{P} \bigl( \bigl\vert X^{(j)} \bigr\vert > n^{\alpha} \bigr) \\ \leq&C\sum_{j=1}^{\infty}\sum _{n=1}^{\infty}n^{\alpha r-1}\sum _{m=n}^{\infty}\mathbb{P} \bigl(m^{\alpha}< \bigl\vert X^{(j)} \bigr\vert \leq (m+1)^{\alpha} \bigr) \\ =&C\sum_{j=1}^{\infty}\sum _{m=1}^{\infty}\mathbb{P} \bigl(m^{\alpha}< \bigl\vert X^{(j)} \bigr\vert \leq(m+1)^{\alpha} \bigr)\sum _{n=1}^{m}n^{\alpha r-1} \\ \leq&C\sum_{j=1}^{\infty}\sum _{m=1}^{\infty}m^{\alpha r}\mathbb {P} \bigl(m^{\alpha}< \bigl\vert X^{(j)} \bigr\vert \leq(m+1)^{\alpha} \bigr) \\ \leq& C\sum_{j=1}^{\infty} \mathbb{E} \bigl\vert X^{(j)} \bigr\vert ^{r}< \infty. \end{aligned}$$

To estimate $I_{2}$, we first show that

$$ n^{-\alpha}\max_{1\leq k\leq n} \Biggl\Vert \sum _{i=1}^{k}a_{ni}\mathbb{E}U_{i} \Biggr\Vert \rightarrow0\quad \text{as }n\rightarrow\infty. $$

Note by the Hölder inequality that $\sum_{i=1}^{n}a_{ni}=O(n)$. So we have by the zero mean assumption, if $\alpha r>1$,

$$\begin{aligned} n^{-\alpha}\max_{1\leq k\leq n} \Biggl\Vert \sum _{i=1}^{k}a_{ni}\mathbb{E}U_{i} \Biggr\Vert =&n^{-\alpha}\max_{1\leq k\leq n} \Biggl\Vert \sum _{i=1}^{k}a_{ni} \mathbb{E}V_{i} \Biggr\Vert \\ \leq&n^{-\alpha}\sum_{j=1}^{\infty}\sum _{i=1}^{n}a_{ni}\mathbb {E} \bigl\vert X_{i}^{(j)} \bigr\vert I\bigl( \bigl\vert X_{i}^{(j)} \bigr\vert >n^{\alpha}\bigr) \\ \leq&Cn^{1-\alpha}\sum_{j=1}^{\infty} \mathbb {E} \bigl\vert X^{(j)} \bigr\vert I\bigl( \bigl\vert X^{(j)} \bigr\vert >n^{\alpha}\bigr) \\ \leq&Cn^{1-\alpha r}\sum_{j=1}^{\infty} \mathbb {E} \bigl\vert X^{(j)} \bigr\vert ^{r}I\bigl( \bigl\vert X^{(j)} \bigr\vert >n^{\alpha}\bigr)\rightarrow0\quad \text{as }n\rightarrow\infty; \end{aligned}$$

and, if $\alpha r=1$, the conclusion above also remains true by the dominated convergence theorem. Therefore, when *n* is large enough,

2 $$ \max_{1\leq k\leq n} \Biggl\Vert \sum_{i=1}^{k}a_{ni}EU_{i} \Biggr\Vert \leq n^{-\alpha}/2. $$

Noting that $\{a_{ni}U_{i}^{(j)},1\leq i\leq n,n\geq1\}$ is NA for any $j\geq1$, one can see that $\{a_{ni}(U_{i}-\mathbb{E}U_{i}),1\leq i\leq n,n\geq1\}$ is CNA. Hence, we have by the Markov inequality, Lemmas 2.1 and 2.2, $\sum_{i=1}^{n}a_{ni}^{2}=O(n)$ and (2)

$$\begin{aligned} I_{2} \leq&C\sum_{n=1}^{\infty}n^{\alpha r-2} \mathbb{P} \Biggl(\max_{1\leq k\leq n} \Biggl\Vert \sum _{i=1}^{k}a_{ni}(U_{i}- \mathbb{E}U_{i}) \Biggr\Vert >\varepsilon n^{\alpha }/2 \Biggr) \\ \leq&C\sum_{n=1}^{\infty}n^{\alpha r-2\alpha-2} \mathbb{E} \Biggl(\max_{1\leq k\leq n} \Biggl\Vert \sum _{i=1}^{k}a_{ni}(U_{i}-\mathbb {E}U_{i}) \Biggr\Vert \Biggr)^{2} \\ \leq&C\sum_{n=1}^{\infty}n^{\alpha r-2\alpha-2}\sum _{i=1}^{n}a_{ni}^{2} \mathbb{E}\|U_{i}-\mathbb{E}U_{i}\|^{2} \\ \leq&C\sum_{n=1}^{\infty}n^{\alpha r-2\alpha-2}\sum _{i=1}^{n}a_{ni}^{2} \mathbb{E}\|U_{i}\|^{2} \\ \leq&C\sum_{j=1}^{\infty}\sum _{n=1}^{\infty}n^{\alpha r-2\alpha -2}\sum _{i=1}^{n}a_{ni}^{2}\mathbb{E} \bigl\vert U_{i}^{(j)} \bigr\vert ^{2} \\ \leq&C\sum_{j=1}^{\infty}\sum _{n=1}^{\infty}n^{\alpha r-2}\sum _{i=1}^{n}a_{ni}^{2}\mathbb{P} \bigl( \bigl\vert X^{(j)} \bigr\vert >n^{\alpha}\bigr) \\ &{}+C\sum_{j=1}^{\infty}\sum _{n=1}^{\infty}n^{\alpha r-2\alpha -2}\sum _{i=1}^{n}a_{ni}^{2}\mathbb{E} \bigl\vert X^{(j)} \bigr\vert ^{2}I\bigl( \bigl\vert X^{(j)} \bigr\vert \leq n^{\alpha}\bigr) \\ \leq&C\sum_{j=1}^{\infty}\sum _{n=1}^{\infty}n^{\alpha r-1}\mathbb {P}\bigl( \bigl\vert X^{(j)} \bigr\vert >n^{\alpha}\bigr) \\ &{}+C\sum_{j=1}^{\infty}\sum _{n=1}^{\infty}n^{\alpha r-2\alpha -1}\mathbb{E} \bigl\vert X^{(j)} \bigr\vert ^{2}I\bigl( \bigl\vert X^{(j)} \bigr\vert \leq n^{\alpha}\bigr) \\ =:&I_{21}+I_{22}. \end{aligned}$$

Similar to the proof of $I_{1}<\infty$, we have $I_{21}<\infty$. Finally, we will estimate $I_{22}$. It is easy to see that

$$\begin{aligned} I_{22} \leq&C\sum_{j=1}^{\infty}\sum _{n=1}^{\infty }n^{\alpha r-2\alpha-1}\mathbb{E} \bigl\vert X^{(j)} \bigr\vert ^{2}I\bigl( \bigl\vert X^{(j)} \bigr\vert \leq n^{\alpha}\bigr) \\ =&C\sum_{j=1}^{\infty}\sum _{n=1}^{\infty}n^{\alpha r-2\alpha -1}\sum _{m=1}^{n}\mathbb{E} \bigl\vert X^{(j)} \bigr\vert ^{2}I\bigl((m-1)^{\alpha }< \bigl\vert X^{(j)} \bigr\vert \leq m^{\alpha}\bigr) \\ =&C\sum_{j=1}^{\infty}\sum _{m=1}^{\infty}\mathbb {E} \bigl\vert X^{(j)} \bigr\vert ^{2}I\bigl((m-1)^{\alpha}< \bigl\vert X^{(j)} \bigr\vert \leq m^{\alpha}\bigr)\sum _{n=m}^{\infty}n^{\alpha r-2\alpha-1} \\ \leq&C\sum_{j=1}^{\infty}\sum _{m=1}^{\infty}m^{\alpha r-2\alpha }\mathbb{E} \bigl\vert X^{(j)} \bigr\vert ^{2}I\bigl((m-1)^{\alpha}< \bigl\vert X^{(j)} \bigr\vert \leq m^{\alpha }\bigr) \\ \leq& C\sum _{j=1}^{\infty}\mathbb{E} \bigl\vert X^{(j)} \bigr\vert ^{r}< \infty. \end{aligned}$$

The proof is completed. □

### Remark 3.1

Theorem 3.1 concerns the weighted sums of random vectors in Hilbert space. If we take $a_{ni}=1$ for any $1\leq i\leq n$, $n\geq1$, the result is still stronger than the corresponding one of Huan et al. [1] since the case $\alpha r=1$ was not considered in Huan et al. [1]; Huan [13] considered the case $\alpha r=1$ for the partial sums of random vectors in Hilbert space, but $1< r<2$ was assumed in that paper. Therefore, Theorem 3.1 improves the corresponding results of Huan et al. [1] and Huan [13], respectively.

### Theorem 3.2

*Let* $1\leq r<2$. *Let* $\{ a_{n},n\geq1\}$ *be a sequence of real numbers such that* $\sum_{i=1}^{n}a_{i}^{2}=O(n)$ *and let* $\{X_{n},n\geq1\}$ *be a sequence of zero mean* *H*-*valued CNA random vectors*. *If* $\{X_{n},n\geq1\}$ *is coordinatewise weakly upper bounded by a random vector* *X*, *then* $\sum_{j=1}^{\infty}\mathbb{E}|X^{(j)}|^{r}<\infty$ *implies that*

$$ \frac{1}{n^{1/r}} \Biggl\Vert \sum_{i=1}^{n}a_{i}X_{i} \Biggr\Vert \rightarrow0\quad \textit{a.s.} $$

### Proof

Applying Theorem 3.1 with $a_{ni}=a_{i}$, for each $1\leq i\leq n$, $n\geq1$ and $\alpha=1/r$, we have, for any $\varepsilon>0$,

$$\begin{aligned} \infty >&\sum_{n=1}^{\infty}n^{-1} \mathbb{P} \Biggl(\max_{1\leq k\leq n} \Biggl\Vert \sum _{i=1}^{k}a_{i}X_{i} \Biggr\Vert >\varepsilon n^{1/r} \Biggr) \\ =&\sum_{m=0}^{\infty}\sum _{n=2^{m}}^{2^{m+1}-1}n^{-1}\mathbb {P} \Biggl(\max _{1\leq k\leq n} \Biggl\Vert \sum_{i=1}^{k}a_{i}X_{i} \Biggr\Vert >\varepsilon n^{1/r} \Biggr) \\ \geq&\frac{1}{2}\sum_{m=0}^{\infty} \mathbb{P} \Biggl(\max_{1\leq k\leq2^{m}} \Biggl\Vert \sum _{i=1}^{k}a_{i}X_{i} \Biggr\Vert >\varepsilon \bigl(2^{m+1}\bigr)^{1/r} \Biggr), \end{aligned}$$

which together with the Borel–Cantelli lemma shows that, as $m\rightarrow\infty$,

$$ \frac{1}{(2^{m})^{1/r}}\max_{1\leq k\leq 2^{m+1}} \Biggl\Vert \sum _{i=1}^{k}a_{i}X_{i} \Biggr\Vert \rightarrow0\quad \mbox{a.s.} $$

Noting that, for any fixed *n*, there exists a positive integer *m* such that $2^{m}\leq n<2^{m+1}$, we have

$$ \frac{1}{n^{1/r}} \Biggl\Vert \sum_{i=1}^{n}a_{i}X_{i} \Biggr\Vert \leq\frac{1}{(2^{m})^{1/r}}\max_{1\leq k\leq2^{m+1}} \Biggl\Vert \sum_{i=1}^{k}a_{i}X_{i} \Biggr\Vert \rightarrow0\quad \mbox{a.s.} $$

The proof is completed. □

### Theorem 3.3

*Let* $1\leq r<2$ *and* $\alpha r\geq 1$. *Let* $\{a_{ni},1\leq i\leq n,n\geq1\}$ *be an array of real numbers such that* $\sum_{i=1}^{n}a_{ni}^{2}=O(n)$. *Let* $\{X_{n},n\geq1\}$ *be a sequence of zero mean* *H*-*valued CNA random vectors*. *Suppose that* $\{ X_{n},n\geq1\}$ *is coordinatewise weakly upper bounded by a random vector* *X*. *Assume that one of the following assumptions holds*:

(i) $\sum_{j=1}^{\infty}\mathbb{E}|X^{(j)}|^{r}<\infty$ *if* $0< q< r$;
(ii) $\sum_{j=1}^{\infty}\mathbb{E}|X^{(j)}|^{r}\log |X^{(j)}|<\infty$ *if* $q=r$;
(iii) $\sum_{j=1}^{\infty}\mathbb{E}|X^{(j)}|^{q}<\infty$ *if* $r< q<2$.

*Then*

$$ \sum_{n=1}^{\infty}n^{\alpha r-\alpha q-2}\mathbb {E} \Biggl(\max_{1\leq k\leq n} \Biggl\Vert \sum _{i=1}^{k}a_{ni}X_{i} \Biggr\Vert -\varepsilon n^{\alpha} \Biggr)_{+}^{q}< \infty. $$

### Proof

From Theorem 3.1 and Lemma 2.3 we can see that

$$\begin{aligned}& \sum_{n=1}^{\infty}n^{\alpha r-\alpha q-2}\mathbb {E} \Biggl(\max_{1\leq k\leq n} \Biggl\Vert \sum _{i=1}^{k}a_{ni}X_{i} \Biggr\Vert -\varepsilon n^{\alpha} \Biggr)_{+}^{q} \\& \quad = \sum_{n=1}^{\infty}n^{\alpha r-\alpha q-2} \int_{0}^{\infty }\mathbb{P} \Biggl(\max _{1\leq k\leq n} \Biggl\Vert \sum_{i=1}^{k}a_{ni}X_{i} \Biggr\Vert -\varepsilon n^{\alpha}>t^{1/q} \Biggr)\,dt \\& \quad = \sum_{n=1}^{\infty}n^{\alpha r-\alpha q-2} \int_{0}^{n^{\alpha q}}\mathbb{P} \Biggl(\max _{1\leq k\leq n} \Biggl\Vert \sum_{i=1}^{k}a_{ni}X_{i} \Biggr\Vert -\varepsilon n^{\alpha}>t^{1/q} \Biggr)\,dt \\& \qquad {} +\sum_{n=1}^{\infty}n^{\alpha r-\alpha q-2} \int_{n^{\alpha q}}^{\infty}\mathbb{P} \Biggl(\max _{1\leq k\leq n} \Biggl\Vert \sum_{i=1}^{k}a_{ni}X_{i} \Biggr\Vert -\varepsilon n^{\alpha}>t^{1/q} \Biggr)\,dt \\& \quad \leq \sum_{n=1}^{\infty}n^{\alpha r-2} \mathbb{P} \Biggl(\max_{1\leq k\leq n} \Biggl\Vert \sum _{i=1}^{k}a_{ni}X_{i} \Biggr\Vert >\varepsilon n^{\alpha} \Biggr) \\& \qquad {} +\sum_{n=1}^{\infty}n^{\alpha r-\alpha q-2} \int_{n^{\alpha q}}^{\infty}\mathbb{P} \Biggl(\max _{1\leq k\leq n} \Biggl\Vert \sum_{i=1}^{k}a_{ni}X_{i} \Biggr\Vert >t^{1/q} \Biggr)\,dt \\& \quad < \infty. \end{aligned}$$

The proof is completed. □

### Remark 3.2

As stated in Sect. 1, the corresponding result in Ko [2] is wrongly established when $r=1$. If we take $a_{ni}=1$ for any $1\leq i\leq n$, $n\geq1$, $q=1$, Theorem 3.3 is equivalent to the corresponding one of Ko [2] when $1< r<2$, $\alpha r>1$. The interesting case $\alpha r=1$, which was not considered in Ko [2], is also considered here. Consequently, Theorem 3.3 generalizes and improves the corresponding result of Ko [2].

### Theorem 3.4

*Suppose that the conditions of Theorem *3.3 *hold with* $\alpha r>1$, *we have*

$$ \sum_{n=1}^{\infty}n^{\alpha r-2}\mathbb{E} \Biggl(\sup_{k\geq n}k^{-\alpha} \Biggl\Vert \sum _{i=1}^{k}a_{ni}X_{i} \Biggr\Vert -\varepsilon \Biggr)_{+}^{q}< \infty. $$

### Proof

By standard calculation, we obtain from Theorem 3.3

$$\begin{aligned}& \sum_{n=1}^{\infty}n^{\alpha r-2}\mathbb{E} \Biggl(\sup_{k\geq n}k^{-\alpha} \Biggl\Vert \sum _{i=1}^{k}a_{ni}X_{i} \Biggr\Vert -\varepsilon \Biggr)_{+}^{q} \\& \quad = \sum_{m=1}^{\infty}\sum _{n=2^{m-1}}^{2^{m}-1}n^{\alpha r-2}\mathbb{E} \Biggl(\sup _{k\geq n}k^{-\alpha} \Biggl\Vert \sum _{i=1}^{k}a_{ni}X_{i} \Biggr\Vert -\varepsilon \Biggr)_{+}^{q} \\& \quad \leq C\sum_{m=1}^{\infty}2^{m(\alpha r-1)} \mathbb{E} \Biggl(\sup_{k\geq2^{m-1}}k^{-\alpha} \Biggl\Vert \sum _{i=1}^{k}a_{ni}X_{i} \Biggr\Vert -\varepsilon \Biggr)_{+}^{q} \\& \quad \leq C\sum_{l=1}^{\infty}\mathbb{E} \Biggl(\max_{2^{l-1}\leq k< 2^{l}}k^{-\alpha} \Biggl\Vert \sum _{i=1}^{k}a_{ni}X_{i} \Biggr\Vert -\varepsilon \Biggr)_{+}^{q}\sum _{m=1}^{l}2^{m(\alpha r-1)} \\& \quad \leq C\sum_{l=1}^{\infty}2^{l(\alpha r-1)} \mathbb{E} \Biggl(\max_{2^{l-1}\leq k< 2^{l}}2^{-\alpha(l-1)} \Biggl\Vert \sum _{i=1}^{k}a_{ni}X_{i} \Biggr\Vert -\varepsilon \Biggr)_{+}^{q} \\& \quad \leq C\sum_{l=1}^{\infty}2^{l(\alpha r-\alpha q-1)} \mathbb{E} \Biggl(\max_{1\leq k< 2^{l}} \Biggl\Vert \sum _{i=1}^{k}a_{ni}X_{i} \Biggr\Vert -\varepsilon2^{\alpha(l-1)} \Biggr)_{+}^{q} \\& \quad \leq C\sum_{l=1}^{\infty}n^{\alpha r-\alpha q-2} \mathbb{E} \Biggl(\max_{1\leq k< n} \Biggl\Vert \sum _{i=1}^{k}a_{ni}X_{i} \Biggr\Vert -\varepsilon2^{-\alpha}n^{\alpha} \Biggr)_{+}^{q}< \infty. \end{aligned}$$

The proof is completed. □

## Conclusions

In this paper, we investigate the complete convergence and the complete moment convergence for sequences of coordinatewise negatively associated random vectors in Hilbert spaces. The obtained results in this paper improve and extend the corresponding theorems of Huan et al. [1] as well as correct and improve the corresponding one of Ko [2].
